# Effects of Central Metal Ion on Binuclear Metal Phthalocyanine-Based Redox Mediator for Lithium Carbonate Decomposition

**DOI:** 10.3390/molecules29092034

**Published:** 2024-04-28

**Authors:** Qinghui Yan, Linghui Yan, Haoshen Huang, Zhengfei Chen, Zixuan Liu, Shaodong Zhou, Haiyong He

**Affiliations:** 1 School of Materials Science and Chemical Engineering, Ningbo University, Ningbo 315211, China; 2111086218@nbu.edu.cn; 2 School of Biological and Chemical Engineering, NingboTech University, Ningbo 315100, China; 13685729957@163.com (H.H.); zhengfei.chen@nbt.edu.cn (Z.C.); 3 College of Chemical and Biological Engineering, Zhejiang University, Hangzhou 310058, China; lhyan@zju.edu.cn; 4 Ningbo Institute of Materials Technology and Engineering, Chinese Academy of Sciences, Ningbo 315201, China; hehaiyong@nimte.ac.cn

**Keywords:** lithium–air batteries, Li_2_CO_3_ decomposition, redox mediator, binuclear metal phthalocyanine, density functional theory

## Abstract

Li_2_CO_3_ is the most tenacious parasitic solid-state product in lithium–air batteries (LABs). Developing suitable redox mediators (RMs) is an efficient way to address the Li_2_CO_3_ issue, but only a few RMs have been investigated to date, and their mechanism of action also remains elusive. Herein, we investigate the effects of the central metal ion in binuclear metal phthalocyanines on the catalysis of Li_2_CO_3_ decomposition, namely binuclear cobalt phthalocyanine (bi-CoPc) and binuclear cobalt manganese phthalocyanine (bi-CoMnPc). Density functional theory (DFT) calculations indicate that the key intermediate peroxydicarbonate (*C_2_O_6_^2−^) is stabilized by bi-CoPc^2+^ and bi-CoMnPc^3+^, which is accountable for their excellent catalytic effects. With one central metal ion substituted by manganese for cobalt, the bi-CoMnPc’s second active redox couple shifts from the second Co(II)/Co(III) couple in the central metal ion to the Pc(-2)/Pc(-1) couple in the phthalocyanine ring. In artificial dry air (N_2_-O_2_, 78:22, *v*/*v*), the LAB cell with bi-CoMnPc in electrolyte exhibited 261 cycles under a fixed capacity of 500 mAh g^−1^_carbon_ and current density of 100 mA g^−1^_carbon_, significantly better than the RM-free cell (62 cycles) and the cell with bi-CoPc (193 cycles).

## 1. Introduction

Along with the fast-paced electrification, intelligentization, and mobilization of industry and daily life, the demand for energy storage devices with higher energy density becomes increasingly urgent. Lithium–air batteries (LABs) possess the highest theoretical energy density (~11,400 Wh/kg, excluding O_2_ mass) among all electrochemical energy storage technologies and have drawn tremendous attention in the past two decades. However, major challenges must be conquered before the practicality of LABs, originating from the highly reactive cathode/anode materials and (intermediate) products (such as metallic lithium, ^1^O_2_, LiO_2_, O_2_^−^·, etc.), as well as the unstable solid–solid contact between discharge/parasitic reaction products and the air cathode [1,2,3,4].

In LABs, Li_2_CO_3_ is regarded as the “Achilles’ heel” [5] because it is both virtually inevitable to form and difficult to remove. On the one hand, Li_2_CO_3_ can be produced both from the absorption of adventitious CO_2_ and from the degradation of cell components (electrolyte solvents, lithium salts, carbon electrodes, etc.). On the other hand, Li_2_CO_3_ is the most tenacious parasitic solid-state product in LABs due to its high oxidative decomposition potential, low electronic/ionic conductivity, and low solubility in most organic solvents. Containing O_2_ in the battery system and converting LABs to lithium–oxygen batteries (LOBs) can mitigate Li_2_CO_3_ accumulation, but at the cost of greatly reduced energy density (~3450 Wh/kg in theory, including O_2_ mass). Therefore, it is necessary to develop suitable catalysts for Li_2_CO_3_ decomposition to enable the utilization of ambient air, especially the redox mediators (RMs) that can overcome the solid–solid contact difficulty between solid-state Li_2_CO_3_ and an air cathode.

Since the first discovery of Li_2_CO_3_ decomposing RM (a planar binuclear cobalt phthalocyanine, bi-CoPc) [6], several other RMs have been reported effective towards Li_2_CO_3_ decomposition, such as LiBr [7,8], conjugated cobalt polyphthalocyanine (CoPPc) [9], cobalt tetramethoxyphenyl porphyrin (CoTMPP) [10], ruthenocene (Ruc) [11], (*1R,2R*)-(-)-*N,N*-bis(3,5-di-*t*-butylsalicylidene)-1,2-cyclohexanediaminocobalt(II) (salen-Co(II)) [12], *N,N,N′,N′*-tetramethyl-*p*-phenylenediamine (TMPD) [13], 5,10-dimethylphenazine (DMPZ) [13], S-phenyl carbonothioate (SPC^−^) [14], 2,5-di-*tert*-butyl-1,4-dimethoxybenzene (DBDMB) [15], etc. However, the Li_2_CO_3_ decomposition mechanism remains elusive. Ideally, Li_2_CO_3_ should decompose via Equation (1), which reverses the discharge reaction to reproduce O_2_ and CO_2_; however, numerous investigations have reported substantially less-than-stoichiometric O_2_ in the evolving gas due to the undesirable decomposition paths (Equations (2) and (3)) and following ^1^O_2_/O_2_^−^ attacks on electrolyte components [16,17,18,19]. Furthermore, the RMs’ exact mechanism of action is still debated, especially the effectiveness of 1-electron RMs in catalyzing Li_2_CO_3_ decomposition. Our previous work proposed the necessity of 2-electron RMs [6], which is supported by some other 2- or multiple-electron ones, such as LiBr [7,8], CoPPc [9], CoTMPP [10], and SPC^−^ [14]. However, recently, some 1-electron RMs have also been reported to be effective, such as Ruc [11], salen-Co(II) [12], DMDMB [15], TMPD [13], DMPZ [13], etc.

(1) 2Li2CO3−4e−→4Li++2CO2+O23

(2) 2Li2CO3−4e−→4Li++2CO2+O21

(3) $$\;2{\mathrm{Li}}_{2}{\mathrm{CO}}_{3}-3e^{-}\to 4{\mathrm{Li}}^{+}+2{\mathrm{CO}}_{2}+{\;O}_{2}^{-}$$

In this work, a planar binuclear cobalt manganese phthalocyanine (bi-CoMnPc, structure shown in Figure S1) is comparatively tested with bi-CoPc to investigate the effects of central metal ions in binuclear metal phthalocyanines on the catalysis of Li_2_CO_3_ decomposition. Density functional theory (DFT) calculations indicate that the key intermediate peroxydicarbonate (*C_2_O_6_^2−^) is stabilized by bi-CoPc^2+^ and bi-CoMnPc^3+^, which is accountable for their excellent catalytic effects. With one central metal ion substituted by manganese for cobalt, the bi-CoMnPc’s second active redox couple for catalyzing Li_2_CO_3_ decomposition shifts from the second Co(II)/Co(III) couple in the central metal ion to the Pc(−2)/Pc(−1) couple in the phthalocyanine ring. In artificial dry air, the LAB cell with bi-CoMnPc exhibited significantly improved cyclability than the RM-free one and even the cell with bi-CoPc.

## 2. Results and Discussion

**Figure 1:**
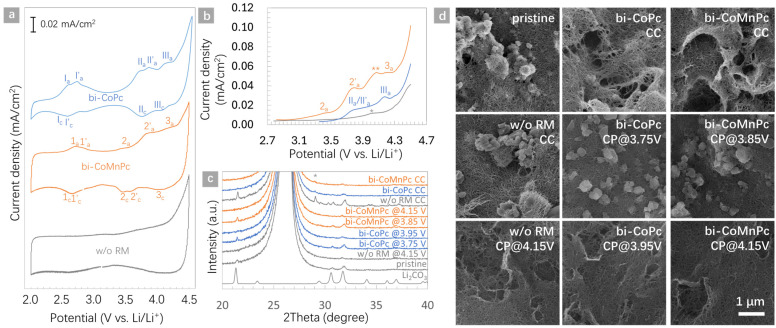
(**a**) Cyclic voltammograms of bi-CoPc and bi-CoMnPc in Ar atmosphere; (**b**) LSV curves of the Li_2_CO_3_-MWCNT composite electrodes with bi-CoMnPc, bi-CoPc, or without RM in the electrolyte, the asterisk (* and **) signs mark the oxidative peaks presumably attributed to Li_2_CO_3_ decomposition; (**c**) XRD patterns of the Li_2_CO_3_-MWCNT composite electrodes after galvanostatic charging (marked as CC) to 4.55 V, or after potentiostatic charging at 3.75/3.95 V vs. Li/Li^+^ with bi-CoPc, at 3.85/4.15 V with bi-CoMnPc, or at 4.15 V without RM, the asterisk (*) sign marks the unknown peak; (**d**) SEM images of the galvanostatically or potentiostatically charged Li_2_CO_3_-MWCNT composite electrodes.

Cyclic voltammetry (CV) in the Ar atmosphere was conducted to identify the electrochemically active redox couples in bi-CoMnPc. As displayed in Figure 1a, bi-CoMnPc shows a pair of redox peaks (marked as 1_a_/1_c_) around 2.69 V vs. Li/Li^+^, with emphasized shoulders (marked as 1′_a_/1′_c_) at slightly higher potentials. These two pairs could be assigned to the Co(I)/Co(II) couple and the Pc(−3)Pc(−2)/Pc(−2)Pc(−2) couple, respectively [6,20−22]. Since their redox potentials are imposed on each other, it is difficult to exactly differentiate them. At around 3.48 V vs. Li/Li^+^, a pair of small peaks are observed, marked as 2_a_/2_c_, which are assigned to the Mn(II)/Mn(III) couple [20,21,22]. Adjacent to that, another pair of redox peaks (marked as 2′_a_/2′_c_) appear at about 3.72 V vs. Li/Li^+^, which is assigned to the Co(II)/Co(III) couple [6,22], similar to that in bi-CoPc (II_a_/II′_a_/II_c_ in Figure 1a). At 3.9~4.2 V vs. Li/Li^+^, a pair of broad peaks (marked as 3_a_/3_c_) are observed in the reductive and oxidative branches, which could be assigned to the redox of phthalocyanine rings, i.e., the Pc(−2)Pc(−1)/Pc(−2)Pc(−2) and Pc(−1)Pc(−1)/Pc(−2)Pc(−1) couples [6,22], similar to that in bi-CoPc (III_a_/III_c_).

According to the function mechanism of RMs, the active redox couples must have higher potential than the target solid-state discharge (or parasitic) product [6,7,8,9,10,11,12,13,14,15], which is 3.71 V vs. Li/Li^+^ for Li_2_CO_3_ (Equation (3)); therefore, in bi-CoMnPc, the active redox couples should be the Co(II)/Co(III), Pc(−2)Pc(−1)/Pc(−2)Pc(−2) and Pc(−1)Pc(−1)/Pc(−2)Pc(−1) couples. Compared to bi-CoPc, due to the substitution of manganese for cobalt, the redox potential of the central metal ion was lowered by ~340 mV from ~3.82 V (with the Co(II)/Co(III) couple) to ~3.48 V (with the Mn(II)/Mn(III) couple). This should have two consequences: on the one hand, the lowered potential would lose the redox couple’s ability to facilitate Li_2_CO_3_ decomposition; on the other hand, it is beneficial to facilitate the decomposition of other solid-state products such as Li_2_O_2_, LiOH, and carboxylates, as reported in previous works [23,24,25].

To investigate the feasibility of bi-CoMnPc for catalyzing Li_2_CO_3_ decomposition, linear scanning voltammetry (LSV), galvanostatic, and potentiostatic charging tests were conducted for Li_2_CO_3_-MWCNT (multiwall carbon nanotube) composite electrodes with bi-CoMnPc, bi-CoPc, or without RM in the electrolyte. As shown in Figure 1b, in the LSV test, without RM in the electrolyte, a small peak (*) emerges at 4.0 V vs. Li/Li^+^, which could be attributed to Li_2_CO_3_ decomposition. When bi-CoPc was added into the electrolyte, the oxidative current density was roughly doubled in the range of 3.75~4.50 V vs. Li/Li^+^, and the oxidative peaks corresponded well to II_a_/II’_a_ and III_a_ as in the Li_2_CO_3_-free CV (Figure 1a). As for the sample with bi-CoMnPc, it is interesting to observe that the oxidative current density was further doubled than that with bi-CoPc. Furthermore, aside from the oxidative peaks 2_a_, 2′_a_, and 3_a_ (as in Figure 1a), a new peak (**) appeared at ~4.05 V vs. Li/Li^+^, which is presumably attributed to Li_2_CO_3_ decomposition. After galvanostatic charging, the preloaded Li_2_CO_3_ was almost completely removed for the samples with bi-CoPc or bi-CoMnPc, leaving cavities in the MWCNT matrix, as characterized by X-ray diffraction (XRD) and scanning electronic microscopy (SEM) test, and shown in Figure 1c,d. In contrast, many Li_2_CO_3_ particles remained in the RM-free sample. Potentiostatic charging tests were also conducted, as shown in Figure 1b–d. After charging with bi-CoMnPc at 3.85 V, most of the preloaded Li_2_CO_3_ still remained in the carbon paper. In sharp contrast, when the charging potential increased to 4.15 V, Li_2_CO_3_ was almost completely removed. For comparison, the same tests were conducted with bi-CoPc at 3.75 V and 3.95 V, respectively. In accordance with our previous work [6], Li_2_CO_3_ remained at 3.75 V but was completely removed at 3.95 V.

Based on these results, it was demonstrated that, similar to bi-CoPc, bi-CoMnPc can also catalyze Li_2_CO_3_ decomposition. However, its catalysis mechanism should be a bit different from that of bi-CoPc because, unlike in bi-CoPc, the second central metal ion (Mn) in bi-CoMnPc is not redox-active for facilitating Li_2_CO_3_ decomposition. In our previous work, it was proposed that the RM-catalyzed Li_2_CO_3_ decomposition requires two electrons extracted from Li_2_CO_3_ to a single RM molecule rather than tandem electron transfer with different RM molecules [6]. Compared to that in bi-CoPc, the absence of the second Co(II)/Co(III) couple obliges the Pc(−2)Pc(−1)/Pc(−2)Pc(−2) couple in bi-CoMnPc to function as the second active redox couple to successfully extract electrons from Li_2_CO_3_ to the RM molecule and subsequently to the working electrode, thus raising the available potential for Li_2_CO_3_ decomposition.

To further investigate the mechanism of RM-catalyzed Li_2_CO_3_ decomposition, we performed DFT calculations for the Li_2_CO_3_ decomposition process with bi-CoPc or bi-CoMnPc. Three possible sets of reaction products, namely CO_2_ and ^3^O_2_, CO_2_ and ^1^O_2_, CO_2_ and O_2_^−^, were considered.

**Figure 2:**
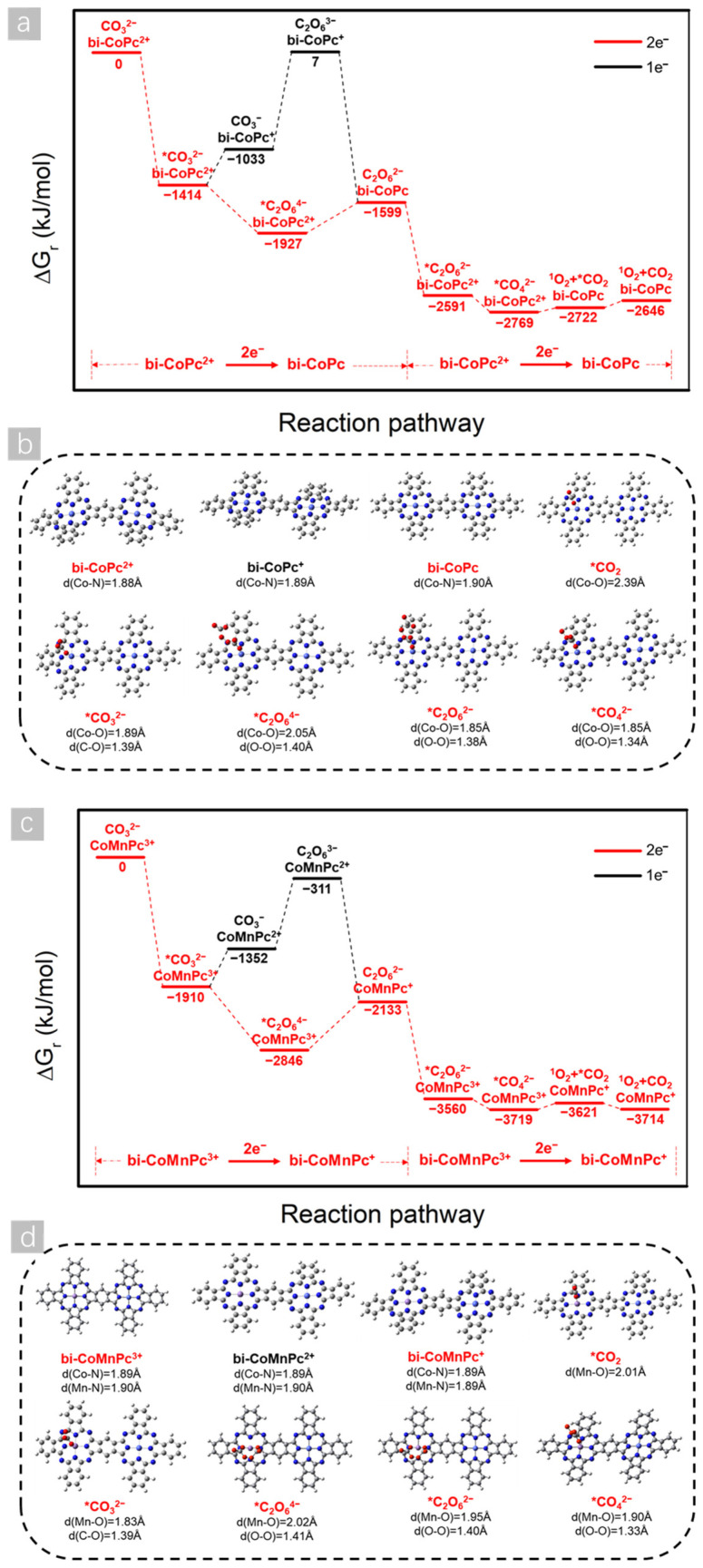
(**a**) The most favorable Li_2_CO_3_ decomposition pathways on bi-CoPc^2+^; (**b**) the corresponding structure evolution of Li_2_CO_3_ decomposition on bi-CoPc^2+^; (**c**) the most favorable Li_2_CO_3_ decomposition pathways on bi-CoMnPc^3+^; (**d**) the corresponding structure evolution of Li_2_CO_3_ decomposition on bi-CoMnPc^3+^.

The most favorable Li_2_CO_3_ decomposition pathways on bi-CoPc^2+^ are illustrated in Figure 2a,b and Equation (4), and the other pathways and corresponding structures are shown in Figures S2–S4. With bi-CoPc^2+^, upon dissociation of Li_2_CO_3_(s) from the solid phase to CO_3_^2−^ and Li^+^, CO_3_^2−^ adsorbs on the bi-CoPc^2+^ with both the O-Co and O-C interactions. Next, another molecular CO_3_^2−^ is adsorbed on the *CO_3_^2−^ structure through O-O interaction to generate *C_2_O_6_^4−^/bi-CoPc^2+^. And then *C_2_O_6_^4−^ loses two electrons to obtain C_2_O_6_^2−^, while bi-CoPc^2+^ receives two electrons to reduce back to neutral bi-CoPc. Compared to the simultaneous transfer of two electrons (*CO_3_^2−^ → *C_2_O_6_^4−^ → C_2_O_6_^2−^ companied by bi-CoPc^2+^ → bi-CoPc), the process of tandem electron transfer, as shown by the black lines in Figure 2a (*CO_3_^2−^ → CO_3_^−^ →C_2_O_6_^3−^ → C_2_O_6_^2−^) is not energetically favorable, regardless of whether the two electrons are gained by bi-CoPc^2+^ → bi-CoPc^+^ → bi-CoPc, 2bi-CoPc^2+^ → 2bi-CoPc^+^ or 2bi-CoPc^+^ → 2bi-CoPc (see Figures S2–S4).

Subsequently, C_2_O_6_^2−^ adsorbs on another bi-CoPc^2+^ and desorbs a CO_2_ molecule to produce *CO_4_^2−^/bi-CoPc^2+^. With an additional two electrons transferred from CO_4_^2−^ to bi-CoPc^2+^, CO_2_ and ^1^O_2_ are accordingly produced, as well as bi-CoPc. Though *CO_4_^2−^ serves as a bifurcation point to lead to three possible sets of reaction products, namely CO_2_ and ^1^O_2_, CO_2_ and ^3^O_2_, and CO_2_ and O_2_^−^, the production of the latter two is kinetically disadvantageous.

For the whole process of Li_2_CO_3_ decomposition, the crucial elementary reaction with the highest reaction energy is *C_2_O_6_^4−^/bi-CoPc^2+^ → C_2_O_6_^2−^/bi-CoPc (∆G_r_ = 328 kJ/mol). Considering both the thermodynamics and kinetics, the main products of Li_2_CO_3_ decomposition correspond to CO_2_ and ^1^O_2_ [6]. The preference for ^1^O_2_ production over ^3^O_2_ and O_2_^−^ may help explain the unusually low O_2_ evolution in Li_2_CO_3_ decomposition compared to that in the Li_2_O_2_ decomposition scenario.

(4) 2Li2CO3+2bi−CoPc2+→4Li++2CO2+O21+2bi−CoPc ∆G1=−2646 kJ/mol

(5) 2Li2CO3+2bi−CoMnPc3+→4Li++2CO2+O21+2bi−CoMnPc3+ ∆G1=−3714 kJ/mol

The reaction pathways for carbonate decomposition on bi-CoMnPc^3+^ are shown in Figure 2c,d and Equation (5), and more detailed information is exhibited in Figures S5–S7. Upon substitution of Mn for Co, more positive charges accumulate on Mn (the NPA charges on Mn and Co in bi-CoMnPc^3+^ are 1.262 and 0.849, respectively). Therefore, the electrostatic interaction between CO_3_^2−^ and the Mn site is slightly stronger than that between CO_3_^2−^ and the Co site, leading to preferable adsorption of CO_3_^2−^ at the manganese site. Although the initial adsorption site of CO_3_^2−^ at bi-CoMnPc^3+^ changes to Mn, the energetically most preferred reaction pathways to form soluble C_2_O_6_^2−^ intermediates are similar (*CO_3_^2−^ → *C_2_O_6_^4−^ → C_2_O_6_^2−^ companies by bi-CoMnPc^3+^ → bi-CoMnPc^+^). Here, the tandem electron transfer to form C_2_O_6_^2−^ accompanied by bi-CoMnPc^3+^ → bi-CoMnPc^2+^ → bi-CoMnPc^+^, 2bi-CoMnPc^3+^ → 2bi-CoMnPc^2+^, or 2bi-CoMnPc^2+^ → 2bi-CoMnPc^+^ are less favorable (see Figures S5–S7). In addition, compared to the decomposition route to generate ^3^O_2_ (Equation (1)) or O_2_^−^ (Equation (3)), the reaction energy for producing ^1^O_2_ (Equation (2)) is also much lower, affording CO_2_ and ^1^O_2_ as the main products with 3714 kJ/mol of energy released for the whole decomposition process, which is much higher than that in the bi-CoPc system (∆G_r_ = -2646 kJ/mol). In this process, the crucial elementary reaction is *C_2_O_6_^4−^/bi-CoMnPc^3+^ → C_2_O_6_^2−^/bi-CoMnPc^+^. Here, the reaction energy for *C_2_O_6_^4−^ → C_2_O_6_^2−^ (∆G_r_ = 713 kJ/mol) is much higher than that in the bi-CoPc system (∆G_r_ = 328 kJ/mol). According to the experimental results, the decomposition voltage of lithium carbonate in the bi-CoMnPc system is higher than that in the bi-CoPc system.

**Scheme 1:**
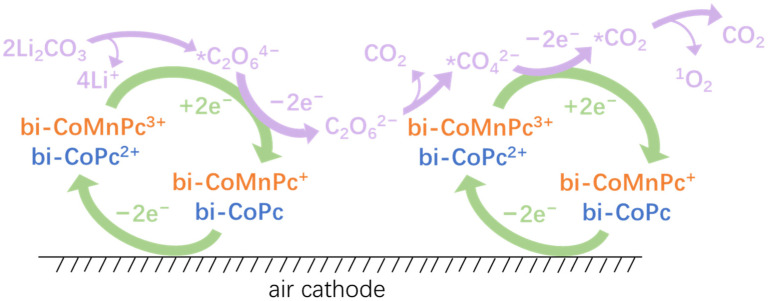
The Li_2_CO_3_ decomposition process involving bi-CoMnPc or bi-CoPc facilitation.

It should be noted that, in the Li_2_CO_3_ decomposition process, the soluble *C_2_O_6_^2−^ is a crucial intermediate because it can desorb and re-adsorb onto another RM molecule (or ion) or separately diffuse onto the air cathode surface to continue decomposition [8,26]. According to the calculation and discussion above, *C_2_O_6_^2−^ is stabilized with the facilitation of bi-CoPc^+^/bi-CoMnPc^+^/bi-CoMnPc^2+^, and even more so with bi-CoPc^2+^/bi-CoMnPc^3+^, favoring *C_2_O_6_^2−^ desorption and subsequent adsorption onto fresh RMs (bi-CoPc^2+^/bi-CoMnPc^3+^) or diffusion onto the air cathode to enhance the decomposition process. It can thus be deduced that, for metal phthalocyanine-type RMs, 2-electron ones are superior to 1-electron ones in facilitating Li_2_CO_3_ decomposition, consistent with our experimental results (Figure 1 and Ref. [6]). And the Li_2_CO_3_ decomposition with the bi-CoMnPc/bi-CoPc facilitation can be illustrated in Scheme 1.

It should also be noted that, other than the oxidative decomposition path, Li_2_CO_3_ could also be removed through the double decomposition reaction (Equation (6)), which has a much lower equilibrium potential (2.80 V vs. Li/Li^+^) than Equations (1)–(3) (3.71–3.82 V). There have been several 1-electron RMs reported effective for catalyzing this reaction, such as manganese phthalocyanine (MnPc) [27], ruthenium acetylacetonate (Ru(acac)_3_) [28], o-phenylenediamine (OPD) [29], phenoxathiine (PHX) [30], etc. Therefore, this reaction should be carefully treated when investigating Li_2_CO_3_ decomposition mechanisms to exclude its interference.

(6) $$2{\mathrm{Li}}_{2}{\mathrm{CO}}_{3}+C-4e^{-}\to \;4{\mathrm{Li}}^{+}+3{\mathrm{CO}}_{2}$$

**Figure 3:**
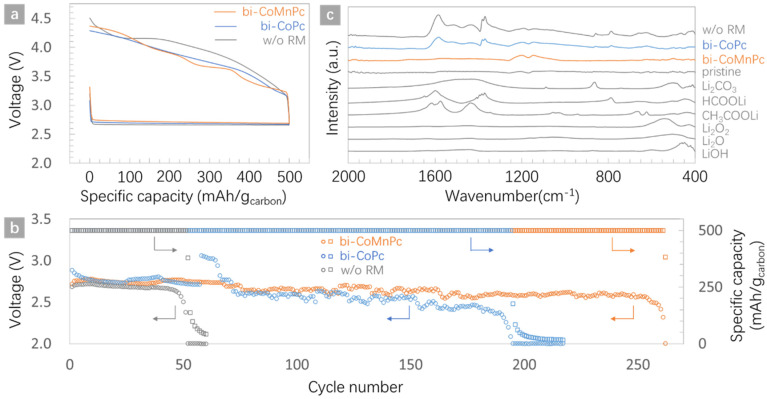
(**a**) The discharge–charge curves of LABs with bi-CoPc, bi-CoMnPc, or without RM in the first cycle, under a current density of 100 mA g^−1^_carbon_ and a fixed capacity of 500 mAh g^−1^_carbon_, voltage range 2.0–4.55 V; (**b**) the evolution of capacity retention and terminal discharge voltage for the LABs with bi-CoPc, bi-CoMnPc, or without RM. The arrows are used to assign the terminal discharge voltage and specific capacity to different vertical axes; (**c**) the FTIR spectra of the air cathodes after 30 discharge–charge cycles with bi-CoPc, bi-CoMnPc, or without RM.

To evaluate the effects of bi-CoMnPc on the cell performance and compare it with bi-CoPc, fixed capacity discharge–charge cycling tests were carried out for LAB coin cells with 2.5 mmol L^−1^ of bi-CoMnPc, with 2.5 mmol L^−1^ of bi-CoPc, or without RM in the electrolyte. The cells were tested in artificial dry air, which is composed of N_2_ and O_2_ (78:22, *v*/*v*). The discharge–charge curves are displayed in Figure 3a. With the addition of bi-CoMnPc, the mid-capacity voltage was reduced by ~260 mV from 4.02 V (without RM) to 3.76 V. This is also ~70 mV lower than that with bi-CoPc, which could be attributed to the lower redox potential of the Mn(II)/Mn(III) couple in bi-CoMnPc than that of the Co(II)/Co(III) couple in bi-CoPc, and thus reduced overpotential for Li_2_O_2_/LiOH decomposition. Aside from the reduction in charging potential, the RMs also exhibited an effect on elevating the discharge voltage; the discharge plateau was raised from 2.66 V without the RM to 2.71 V with bi-CoMnPc and 2.68 V with bi-CoPc. A similar phenomenon has been observed with other metal phthalocyanine complexes, such as mononuclear cobalt phthalocyanine [6] and iron phthalocyanine [31], which could be attributed to their redox mediation effect for the catalysis of oxygen reduction reactions (ORRs).

With RM addition in the electrolyte, the cyclic life was significantly enhanced from 52 cycles (without RM) to 193 cycles (with bi-CoPc) and 261 cycles (with bi-CoMnPc), as shown in Figure 3b. Ex situ Fourier transform infrared (FTIR) spectroscopy in Figure 3c clearly shows that, after 30 cycles of operation, intense signals of Li_2_CO_3_ and lithium carboxylates became observable for the RM-free cell. In contrast, for the cell with bi-CoPc and bi-CoMnPc, the Li_2_CO_3_ and lithium carboxylate peaks are greatly suppressed. It is noted that the Li_2_CO_3_ and lithium carboxylate peaks are even less prominent for the cell with bi-CoMnPc than that with bi-CoPc, indicating bi-CoMnPc’s superior suppression effect on parasitic product accumulation. To further investigate the accumulation status of parasitic products, ex situ energy dispersive spectroscopy (EDS) mapping was used to semi-quantitatively characterize the cycled air cathodes. As shown in Figures S8–S11, the oxygen content in the air cathodes with bi-CoPc or bi-CoMnPc is roughly only half of the RM-free one, indicating their good capabilities in removing parasitic products.

It has been widely perceived that high charging potential is detrimental to the cyclability of LOBs/LABs because it would induce more parasitic reactions, consuming electrolyte/carbon electrodes and accumulating solid products (such as Li_2_CO_3_, lithium carboxylates, and polyesters) on the air cathode [32]. As discussed above, on the one hand, with the substitution of manganese for cobalt, the RM’s functioning potential for Li_2_CO_3_ decomposition is raised from ~3.95 V (with bi-CoPc) to ~4.15 V (with bi-CoMnPc), but on the other hand, its functioning potential for Li_2_O_2_/LiOH decomposition is reduced by ~260 mV from 3.74 V to 3.48 V. As a result of these competing effects, bi-CoMnPc apparently exhibited better performance in improving the cell cyclability, probably because Li_2_O_2_ is still the majority of solid-state discharge products in the CO_2_-free artificial dry air.

## 3. Materials and Methods

### 3.1. Materials

Binuclear cobalt phthalocyanine (bi-CoPc) and binuclear cobalt manganese phthalocyanine (bi-CoMnPc) were purchased from Shanghai Dibai Chemical Technologies Co., Ltd. (Shanghai, China). Tetraethylene glycol dimethyl ether (TEGDME) from Aladdin Scientific (Shanghai, China) was dried for at least 72 h over activated 3 Å molecular sieves. Li_2_CO_3_ was ball milled for 6 h at 400 rpm prior to use. All reagents were used as received unless specified.

### 3.2. Electrochemical Measurements

The cyclic voltammetry (CV), linear sweep voltammetry (LSV), and charging and discharging tests were conducted in CR2032 coin cells. The air cathode was prepared by pasting a slurry of ketjenblack (KB) EC600JD carbon black onto a piece of carbon paper (TGP-H-060, Toray, Tokyo, Japan) with a loading of ∼0.15 mg_carbon_ cm^−2^. Polytetrafluoroethylene (PTFE) was used as the binder, with KB/PTFE = 85:15 (*w*/*w*). The Li_2_CO_3_-preloaded working electrode was prepared by casting a slurry of Li_2_CO_3_-MWCNT-PVDF (4:5:1, *w*/*w/w*) onto a piece of carbon paper with a loading of ∼0.5 mg_carbon_ cm^−2^. A piece of lithium foil (200 μm thick) was used as the anode. The base electrolyte was 1 M lithium bis(trifluoromethanesulfonyl)imide (LiTFSI) in 140 μL of TEGDME. Electrolytes containing bi-CoPc or bi-CoMnPc were prepared by dissolving them into the base electrolyte at a concentration of 2.5 mM. A piece of glass fiber filter (Whatman GF/D, Whatman, Maidstone, UK) and a piece of PP (polypropylene) membrane (Celgard 2400, Celgard, Charlotte, NC, USA) were used as the separators. The coin cells were assembled in an Ar-filled glovebox and relaxed for 12 h, and then the tests were performed either in Ar or in a simulated dry air environment with N_2_/O_2_ (78:22, *v*/*v*). Potentiostatic and galvanostatic charging tests, as well as cycling tests, were conducted using a Neware battery testing system. The current density was set to 100 mA g^−1^_carbon_, with a cutoff voltage of 2.0 V for discharge and 4.50/4.55 V for charge. CV and LSV tests were conducted with a Solartron 1470E electrochemical workstation (Solartron Metrology, Bognor Regis, UK).

### 3.3. Physical Characterizations

Powder X-ray diffractograms (XRD) were obtained on a D8 ADVANCE DaVinci diffractometer (Bruker, Billerica, MA, USA) with Cu Kα radiation (λ = 1.5418 Å). Fourier transform infrared (FTIR) spectra were collected on a Nicolet 6700 spectrometer (Thermo Fisher Scientific, Waltham, MA, USA) in transmission mode or on a Cary 660+620 spectrometer (Agilent, Santa Clara, CA, USA) in attenuated total reflectance (ATR) mode. Scanning electron microscopy (SEM) was performed on a Hitachi S4800 (Hitachi, Tokyo, Japan) unit or on a Quanta 250 FEG unit (FEI, Hillsboro, OR, USA).

### 3.4. Computation Methods

The DFT calculation was performed by using the Gaussian 16 [33], xTB (version 6.3.2) [34,35], and ORCA 5.0 [36] programs. Structural optimization and frequency analysis were performed at the GFN2-xTB level as interfaced into the Gaussian 16 program using the gau_xtb code [37] to obtain the thermal correction to Gibbs energy in the gas phase. Stationary points were optimized without symmetry constraints, and their nature was confirmed by vibrational frequency analysis. All structures given are at minimal points and have no imaginary frequencies. Then, more accurate single-point energies were obtained at the ωB97X [38]-D3BJ [39,40]/def2-TZVPP [41] level with ORCA. The Gibbs free energy G was calculated as G = E (single-point energies obtained by wb97X-D3BJ/def2-TZVPP) + E(thermal correction to Gibbs by GFN2-xTB). And ∆G was calculated by ΔG = ΔE + ΔZPE − TΔS, where ΔE, ΔZPE, T, and ΔS indicated the single-point energy change, zero-point energy change, temperature, and entropy change, respectively. In the decomposition pathways, the intermediate label * represents adsorption on the phthalocyanine structure; otherwise, it means in the gas phase state. The total charge of the structures is considered with the change in the adsorption state, which is the sum of the charges of the binuclear metal phthalocyanine and the adsorption structure. The natural bond orbital (NBO) [42,43,44,45,46,47] calculations were performed to obtain further information.

## 4. Conclusions

The effects of central metal ions in binuclear metal phthalocyanines have been investigated in terms of their catalytic activity towards Li_2_CO_3_ decomposition. DFT calculations indicate that the key intermediate peroxydicarbonate (*C_2_O_6_^2−^) is stabilized by bi-CoPc^2+^ and bi-CoMnPc^2+^, which is accountable for their improved catalytic activity compared to bi-CoPc^+^/bi-CoMnPc^+^ and pristine bi-CoPc/bi-CoMnPc. With one central metal ion substituted by manganese for cobalt, the bi-CoMnPc’s second active redox couple for catalyzing Li_2_CO_3_ decomposition shifts from the second Co(II)/Co(III) couple in the central metal ion to the Pc(-2)/Pc(-1) couple in the phthalocyanine ring. In artificial dry air, the LAB cell with bi-CoMnPc in electrolyte exhibited 261 cycles under a fixed capacity of 500 mAh g^−1^_carbon_ and current density of 100 mA g^−1^_carbon_, significantly better than the RM-free cell (62 cycles) and even the cell with bi-CoPc (193 cycles). We hope this work sheds new insights into the mechanisms of RM-facilitated Li_2_CO_3_ decomposition and designs more efficient Li_2_CO_3_ decomposing RMs.

## Data Availability

The data presented in this study are available in article and Supplementary Materials.

## Supplementary Materials

The following supporting information can be downloaded at https://www.mdpi.com/article/10.3390/molecules29092034/s1, Figure S1: The structures of bi-CoPc and bi-CoMnPc. Figure S2: The Li_2_CO_3_ decomposition pathways on bi-CoPc^2+^ and corresponding structures with bi-CoPc^2+^→bi-CoPc. Figure S3: The Li_2_CO_3_ decomposition pathways on pristine bi-CoPc^2+^ and the corresponding structures with 2bi-CoPc^2+^→2bi-CoPc^+^. Figure S4: The Li_2_CO_3_ decomposition pathways on bi-CoPc^+^ and the corresponding structures with 2bi-CoPc^+^→2bi-CoPc. Figure S5: The Li_2_CO_3_ decomposition pathways on pristine bi-CoMnPc^3+^ and the corresponding structures with bi-CoMnPc^3+^→bi-CoMnPc^+^. Figure S6: The Li_2_CO_3_ decomposition pathways on pristine bi-CoMnPc^3+^ and the corresponding structures with 2bi-CoMnPc^3+^→2bi-CoMnPc^2+^. Figure S7: The Li_2_CO_3_ decomposition pathways on pristine bi-CoMnPc^2+^ and the corresponding structures with 2bi-CoMnPc^2+^→2bi-CoMnPc^+^. Figure S8: The SEM images and EDS mapping results of carbon and oxygen for the air cathode (a) without RM, (b) with bi-CoPc and (c) with bi-CoMnPc after 1 discharge-charge cycle. Figure S9: The SEM images and EDS mapping results of carbon and oxygen for the air cathode (a) without RM, (b) with bi-CoPc and (c) with bi-CoMnPc after 10 discharge-charge cycles. Figure S10: The SEM images and EDS mapping results of carbon and oxygen for the air cathode (a) without RM, (b) with bi-CoPc and (c) with bi-CoMnPc after 30 discharge-charge cycles. Figure S11: The oxygen content in the cycled air cathodes according to EDS results.
