# Supplementary material for: Effects of Central Metal Ion on Binuclear Metal Phthalocyanine-Based Redox Mediator for Lithium Carbonate Decomposition
Source: Molecules. 2024 Apr 28;29(9):2034. doi: 10.3390/molecules29092034 (PMC11085934; doi:10.3390/molecules29092034)
Supplement: Supplementary file 1 [file molecules-29-02034-s001.zip › molecules-2908064-supplementary.pdf]

Supplementary Materials

Effects of Central Metal Ion on Binuclear Metal Phthalocyanine-Based Redox Mediator for Lithium Carbonate Decomposition

Qinghui Yan [1,4,†], Linghui Yan [3,†], Haoshen Huang [2], Zhengfei Chen [2], Zixuan Liu [2,4,*], Shaodong Zhou [3,*] and Haiyong He [4]

[1] School of Materials Science and Chemical Engineering, Ningbo University, Ningbo 315211, China; 2111086218@nbu.edu.cn

[2] School of Biological and Chemical Engineering, NingboTech University, Ningbo 315100, China; 13685729957@163.com (H.H.); zhengfei.chen@nbt.edu.cn (Z.C.)

[3] College of Chemical and Biological Engineering, Zhejiang University, Hangzhou 310058, China; lhyan@zju.edu.cn

[4] Ningbo Institute of Materials Technology and Engineering, Chinese Academy of Sciences, Ningbo 315201, China; hehaiyong@nimte.ac.cn

* Correspondence: liuzixuan@nbt.edu.cn (Z.L.); szhou@zju.edu.cn (S.Z.); Tel.: +86-574-882-295-98 (Z.L.); +86-571-879-516-15 (S.Z.)

† These authors contributed equally to this work.

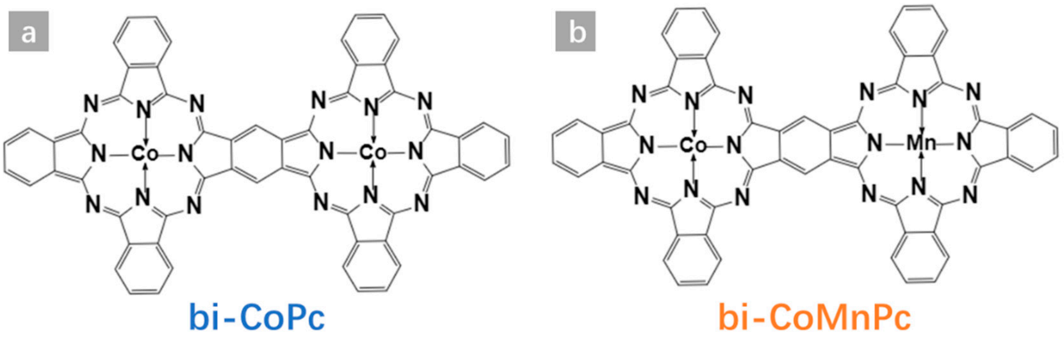

Figure S1. The structures of bi-CoPc and bi-CoMnPc

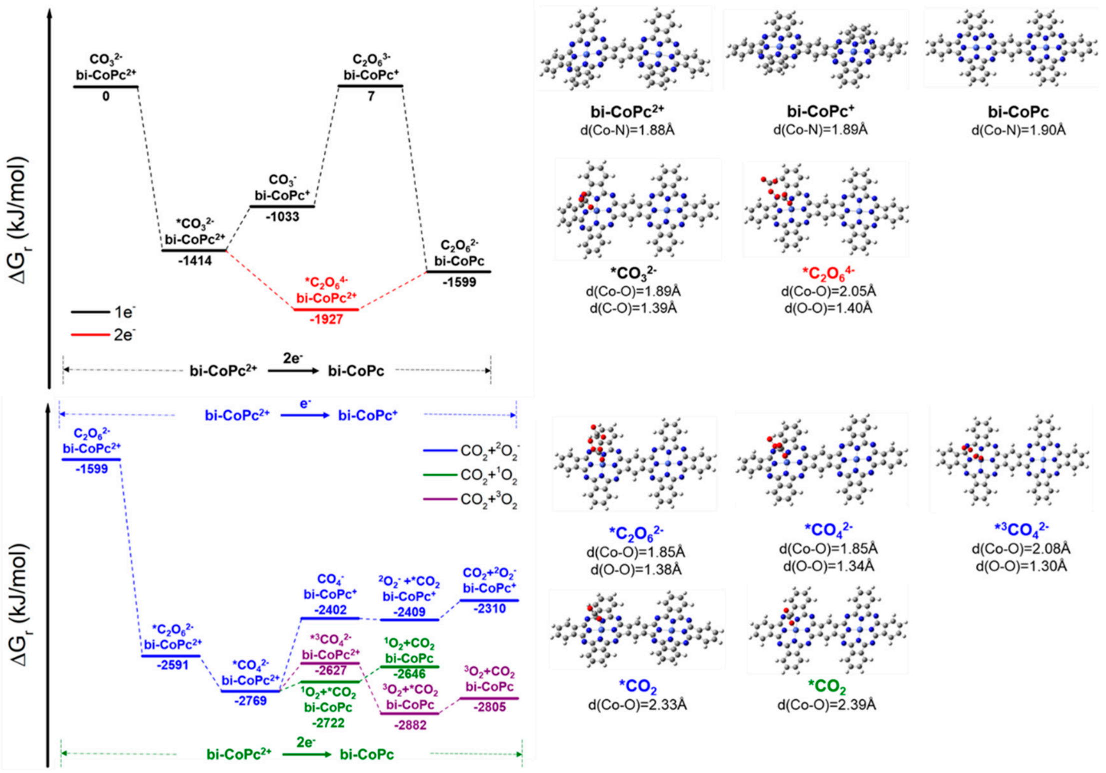

Figure S2. The Li_2CO_3 decomposition pathways on bi-$CoPc^{2+}$ and corresponding structures with bi-$CoPc^{2+}$→bi-CoPc

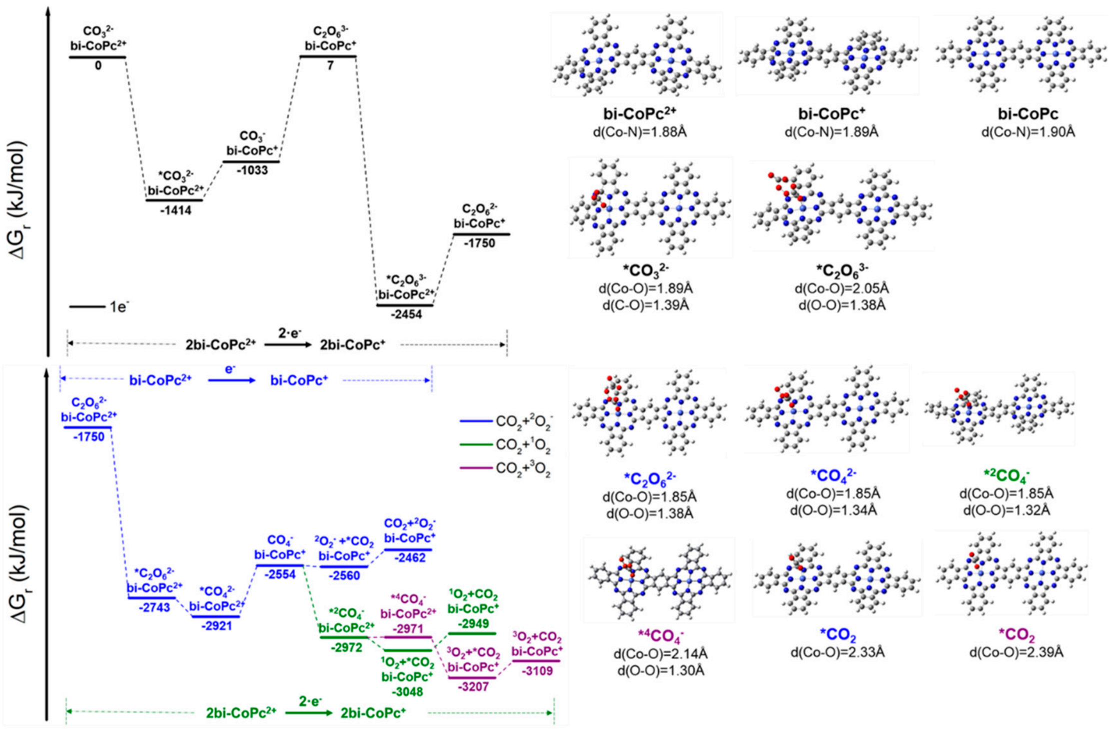

Figure S3. The Li_2CO_3 decomposition pathways on pristine bi-$CoPc^{2+}$ and the corresponding structures with 2bi-$CoPc^{2+}$→2bi-$CoPc^{+}$

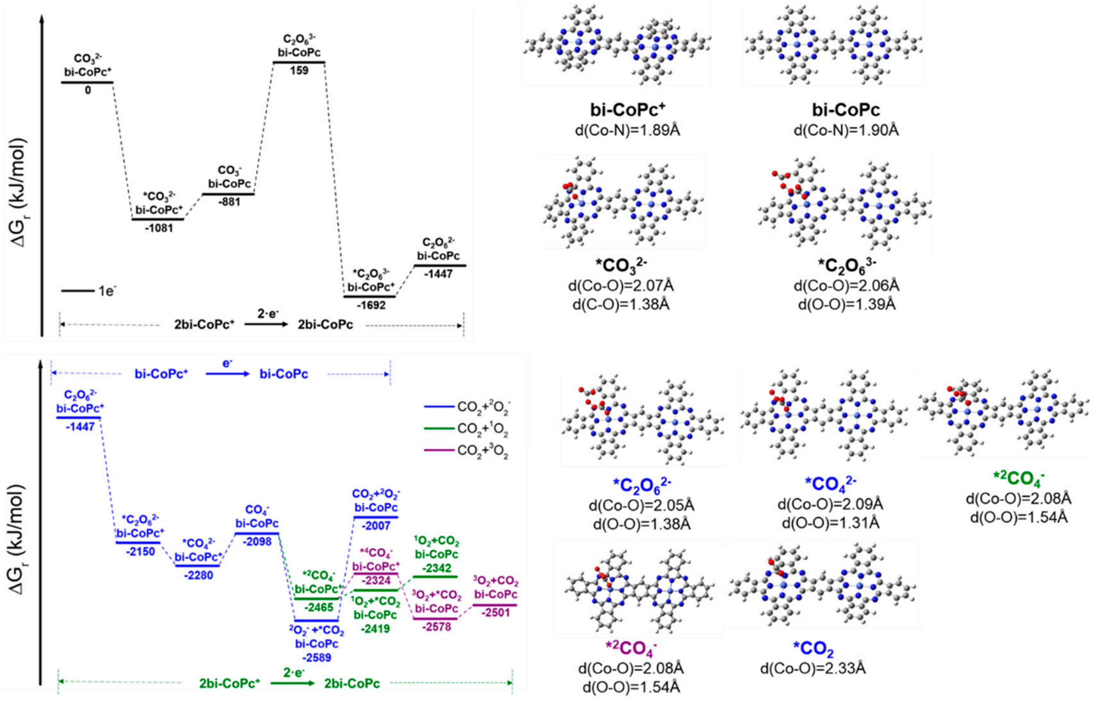

Figure S4. The Li_2CO_3 decomposition pathways on bi-CoPc^{+} and the corresponding structures with 2bi-CoPc^{+}→2bi-CoPc

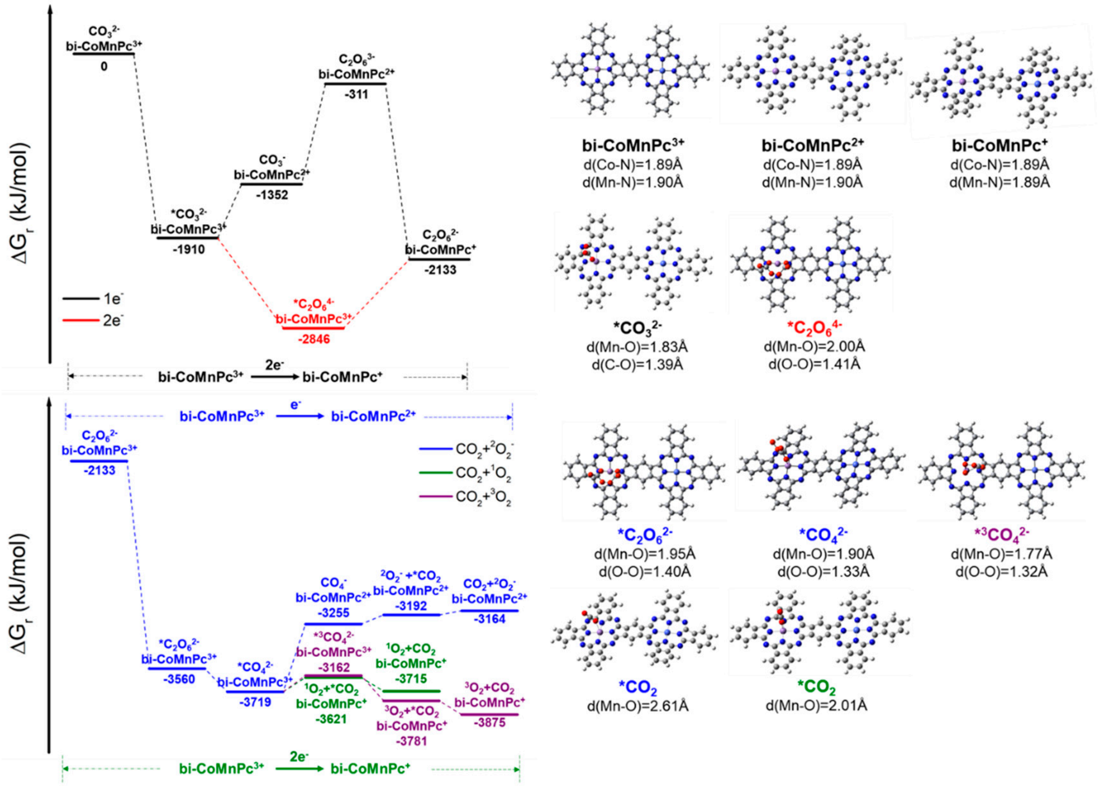

Figure S5. The Li_2CO_3 decomposition pathways on pristine bi-CoMnPc^{3+} and the corresponding structures with bi-CoMnPc^{3+}→bi-CoMnPc^{+}

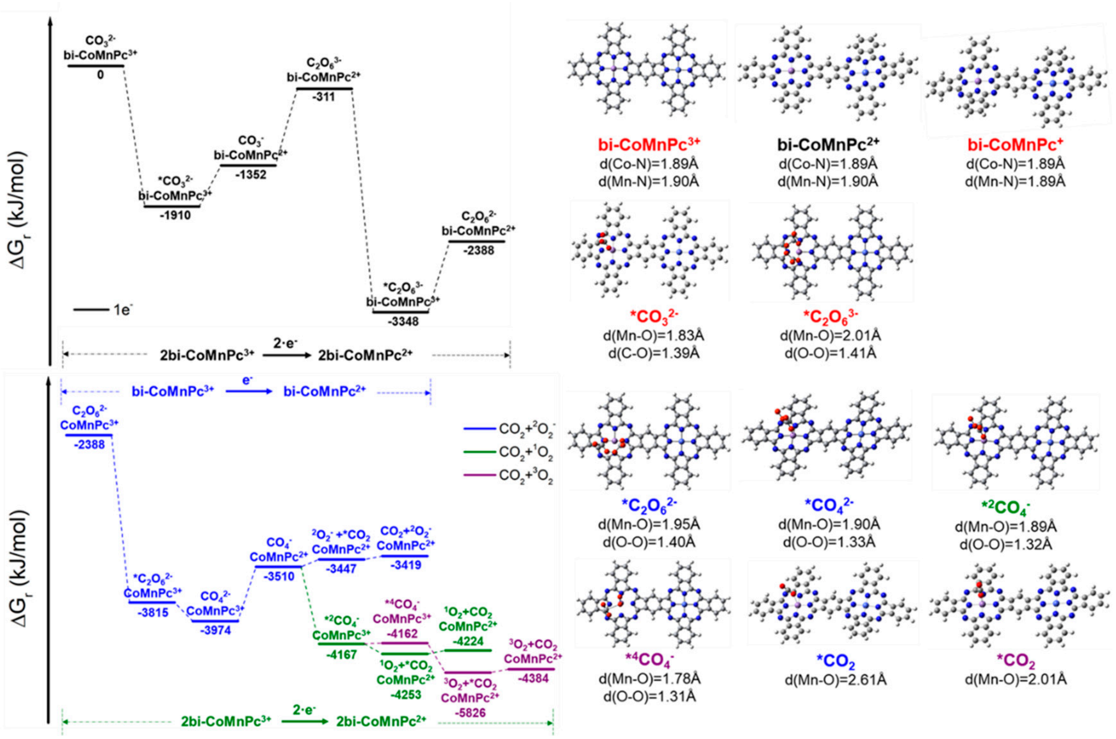

Figure S6. The Li_2CO_3 decomposition pathways on pristine bi-CoMnPc^{3+} and the corresponding structures with 2bi-CoMnPc^{3+}→2bi-CoMnPc^{2+}

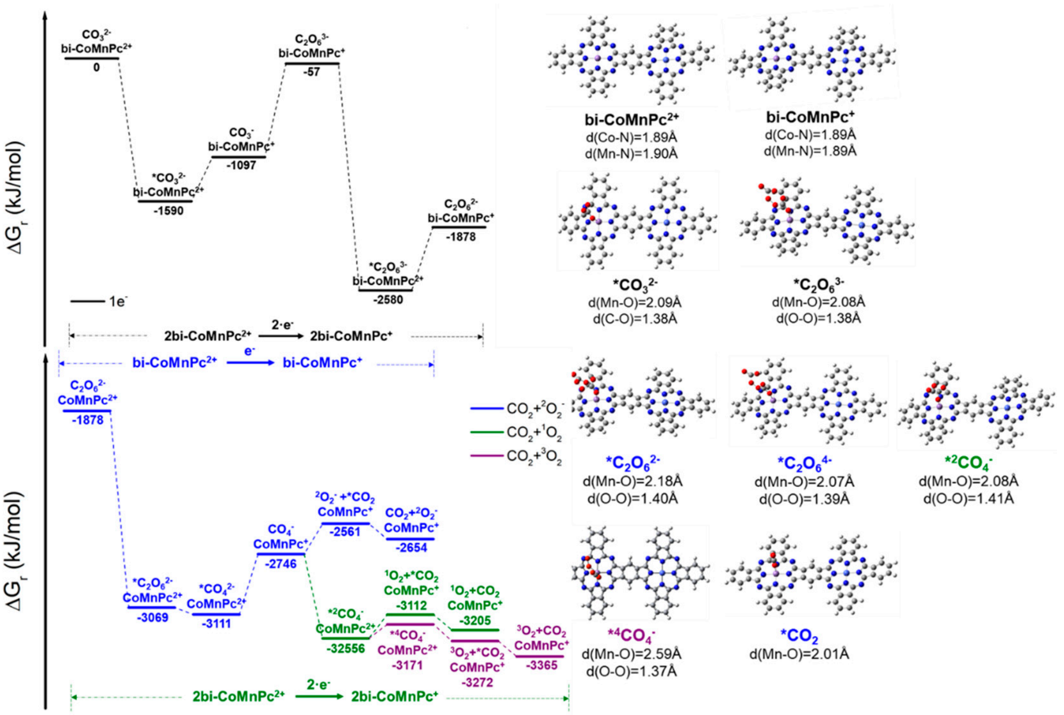

Figure S7. The Li_2CO_3 decomposition pathways on pristine bi-CoMnPc^{2+} and the corresponding structures with 2bi-CoMnPc^{2+}→2bi-CoMnPc^{+}

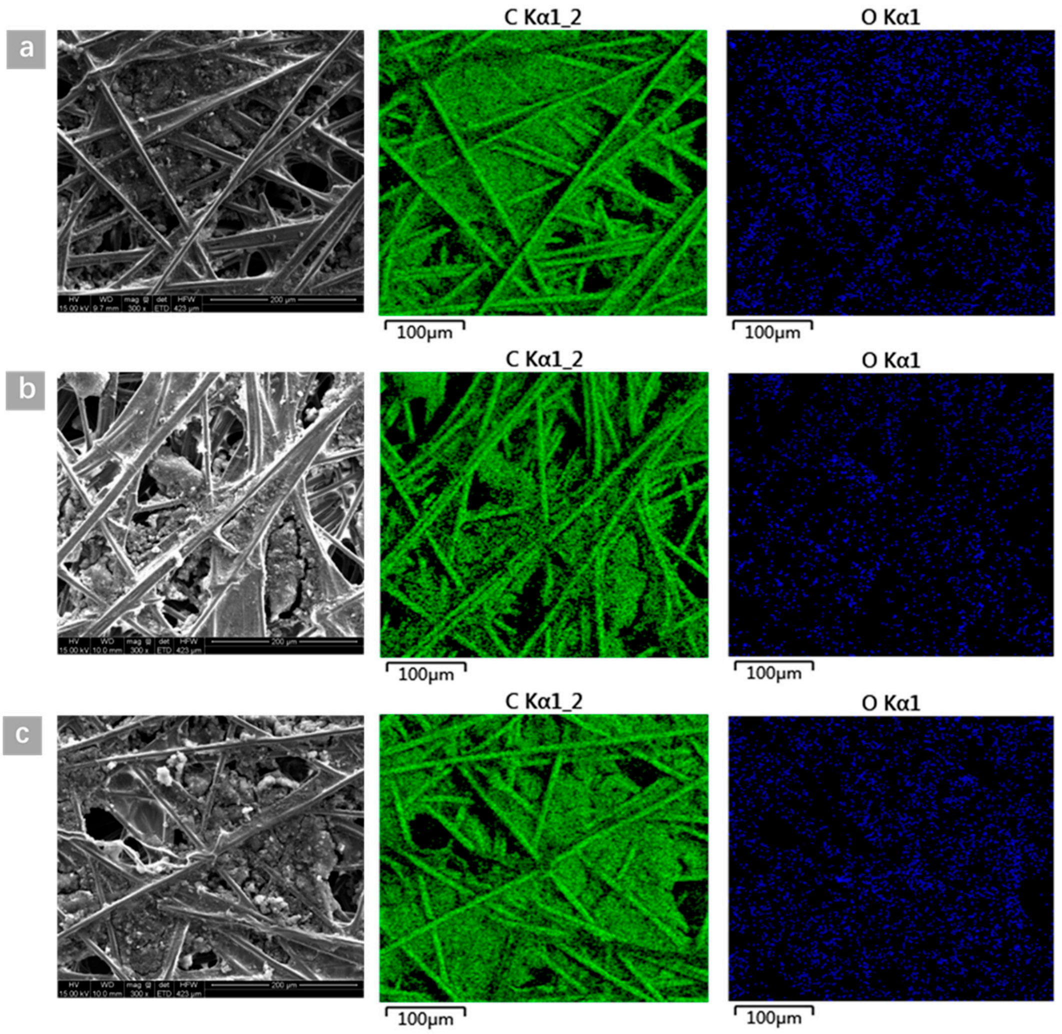

Figure S8. The SEM images and EDS mapping results of carbon and oxygen for the air cathode (a) without RM, (b) with bi-CoPc and (c) with bi-CoMnPc after 1 discharge-charge cycle.

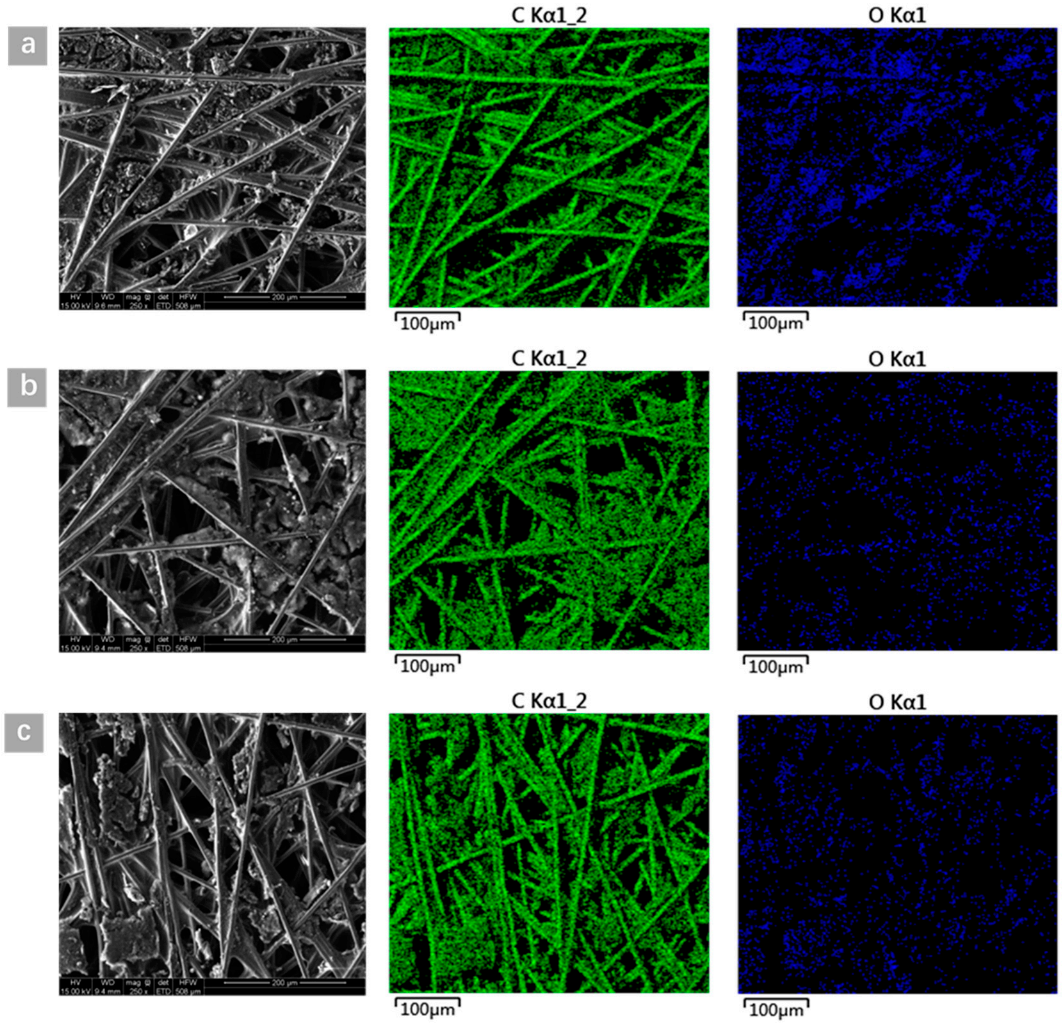

Figure S9. The SEM images and EDS mapping results of carbon and oxygen for the air cathode (a) without RM, (b) with bi-CoPc and (c) with bi-CoMnPc after 10 discharge-charge cycles.

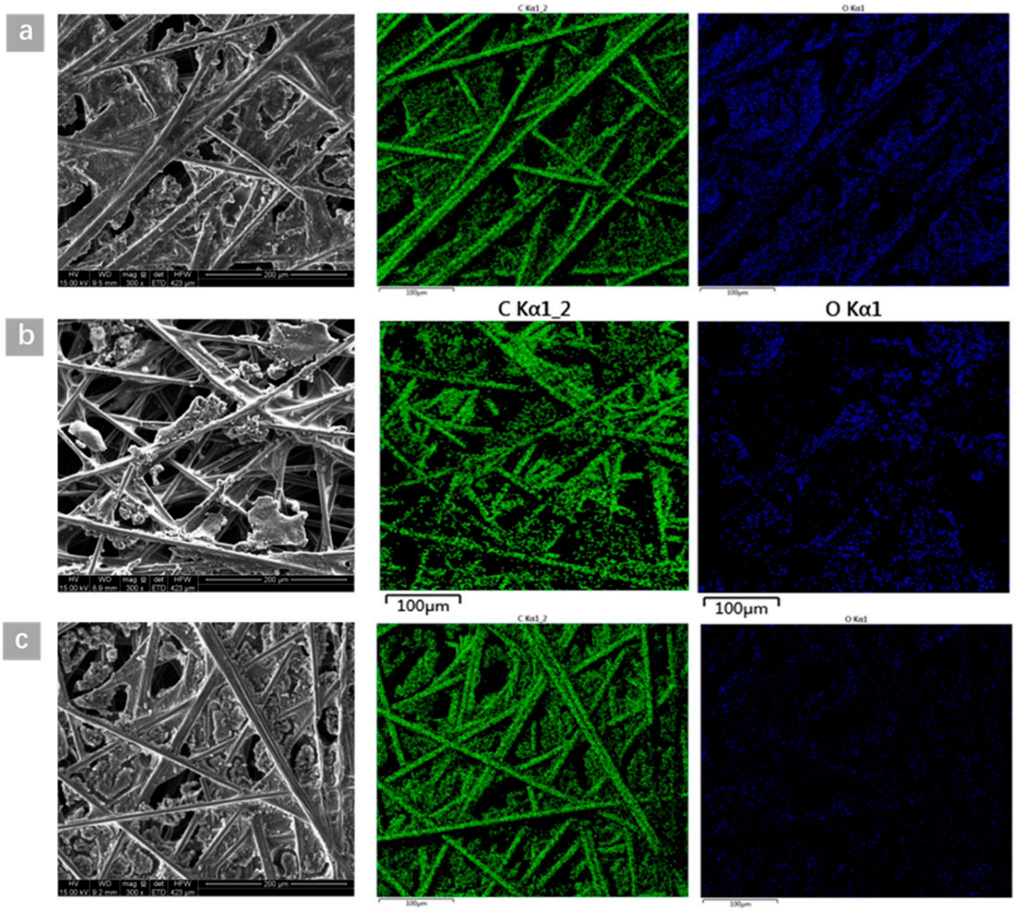

Figure S10. The SEM images and EDS mapping results of carbon and oxygen for the air cathode (a) without RM, (b) with bi-CoPc and (c) with bi-CoMnPc after 30 discharge-charge cycles.

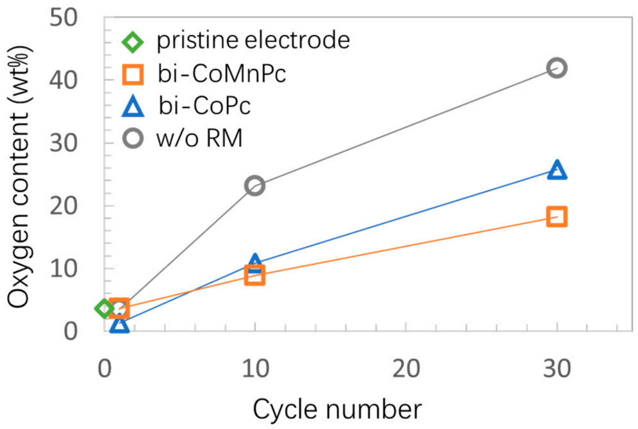

Figure S11. The oxygen content in the cycled air cathodes according to EDS results.
